# Correlation between Ribosome Biogenesis and the Magnitude of Hypertrophy in Overloaded Skeletal Muscle

**DOI:** 10.1371/journal.pone.0147284

**Published:** 2016-01-29

**Authors:** Satoshi Nakada, Riki Ogasawara, Shigeo Kawada, Takahiro Maekawa, Naokata Ishii

**Affiliations:** 1 Department of Life Sciences, Graduate School of Arts and Sciences, The University of Tokyo, Tokyo, Japan; 2 Department of Sport and Medical Science, Teikyo University Faculty of Medical Technology, Tokyo, Japan; University of Louisville School of Medicine, UNITED STATES

## Abstract

External loads applied to skeletal muscle cause increases in the protein translation rate, which leads to muscle hypertrophy. Although some studies have demonstrated that increases in the capacity and efficiency of translation are involved in this process, it remains unclear how these two factors are related to the magnitude of muscle hypertrophy. The present study aimed to clarify the roles played by the capacity and efficiency of translation in muscle hypertrophy. We used an improved synergist ablation in which the magnitude of compensatory hypertrophy could be controlled by partial removal of synergist muscles. Male rats were assigned to four groups in which the plantaris muscle was unilaterally subjected to weak (WK), moderate (MO), middle (MI), and strong (ST) overloading by four types of synergist ablation. Fourteen days after surgery, the weight of the plantaris muscle per body weight increased by 8%, 22%, 32% and 45%, in the WK, MO, MI and ST groups, respectively. Five days after surgery, 18+28S rRNA content (an indicator of translational capacity) increased with increasing overload, with increases of 1.8-fold (MO), 2.2-fold (MI), and 2.5-fold (ST), respectively, relative to non-overloaded muscle (NL) in the WK group. rRNA content showed a strong correlation with relative muscle weight measured 14 days after surgery (r = 0.98). The phosphorylated form of p70S6K (a positive regulator of translational efficiency) showed a marked increase in the MO group, but no further increase was observed with further increase in overload (increases of 22.6-fold (MO), 17.4-fold (MI), and 18.2-fold (ST), respectively, relative to NL in the WK group). These results indicate that increases in ribosome biogenesis at the early phase of overloading are strongly dependent on the amount of overloading, and may play an important role in increasing the translational capacity for further gain of muscular size.

## Introduction

In skeletal muscle, it is generally known that the increase of muscle mass subsequent to application of an external load is achieved by the accumulation of increasing of protein synthesis [1]. Among the processes involved in protein synthesis, protein translation has a central role in determining the amount of protein synthesized.

To ascertain the part played by translation in overload and/or exercise-induced muscle hypertrophy, contributions of the capacity and efficiency of translation must be considered [2]. Both processes have been thought to be important in the exercise-induced increase in protein synthesis. However, most studies have focused on the mechanisms controlling translational efficiency (e.g., ribosome activation through the mammalian target of rapamycin (mTOR) C1 signaling pathway [3,4]) and not on the contribution of “translational capacity”.

Translational capacity is determined by the amount of “translational machinery” per unit volume of cells: ribosome numbers, transfer ribonucleic acid (tRNA) molecules, and translational factors. All three factors are important, but the number of ribosomes present in the cell has been thought to be a primary determinant of translational capacity [5]. Therefore, ribosome biogenesis may have an essential role in the control of protein synthesis and cell growth [6,7]. Involvement of ribosome biogenesis has been shown in the growth of cardiac muscle [5,8–10], but little is known about the contribution of ribosome biogenesis to hypertrophy of skeletal muscle. Recently, some studies have shown increased ribosome content in skeletal muscle hypertrophied by synergist ablation in rats [11–15] and in human skeletal muscle after resistance-exercise training [16]. However whether a quantitative relationship exists between the external loads applied to the muscle and ribosome biogenesis is not known.

“Translational efficiency” is defined as the rate of protein synthesis per ribosome, and is limited mainly by the initiation step of translation. Baar and Esser reported a strong positive correlation between phosphorylation-induced activation of p70S6K (an initiator of translation) and the magnitude of hypertrophy in muscles subjected to mechanical loading [17]. Therefore, p70S6K could be the main regulator of the mass of skeletal muscle. However, more recent studies have shown weak or no correlation between p70s6k phosphorylation and the magnitude of muscle hypertrophy [18–20].

Thus, our aims were: (i) to establish an animal model of muscle hypertrophy in which the magnitude of hypertrophy can be controlled in a stepwise manner; and (ii) to ascertain if the magnitude of muscle hypertrophy is correlated with ribosome biogenesis and/or p70S6K activation in the early phase of overloading.

## Materials and Methods

### Animals

Sixty-four male Wistar rats (11 weeks; 330 g) were purchased from CLEA Japan (Tokyo, Japan). They were housed in individual cages at regulated temperature (22°C), humidity (60%), and illumination cycles (12-h light and 12-h dark). They were allowed to eat commercial rat chow (CE2; CLEA Japan) and drink water *ad libitum*. The health status of the rats was monitored everyday in terms of signs of infections or wound openings. No rats showed any signs of illness or died at any time prior to the experimental endpoint. Rat care and all experimental procedures employed were in accordance with the policy statement of the American College of Sports Medicine on research with experimental animals. The Ethical Committee for Animal Experiments at the University of Tokyo approved this study.

### Overload Surgery

Rats were divided into four weight-matched groups of 16. They were subjected to four types of ablation surgery of synergist muscles: “weak” overload surgery (WK); “moderate” overload surgery (MO); “middle” overload surgery (MI); “strong” overload surgery (ST).

**Fig 1:**
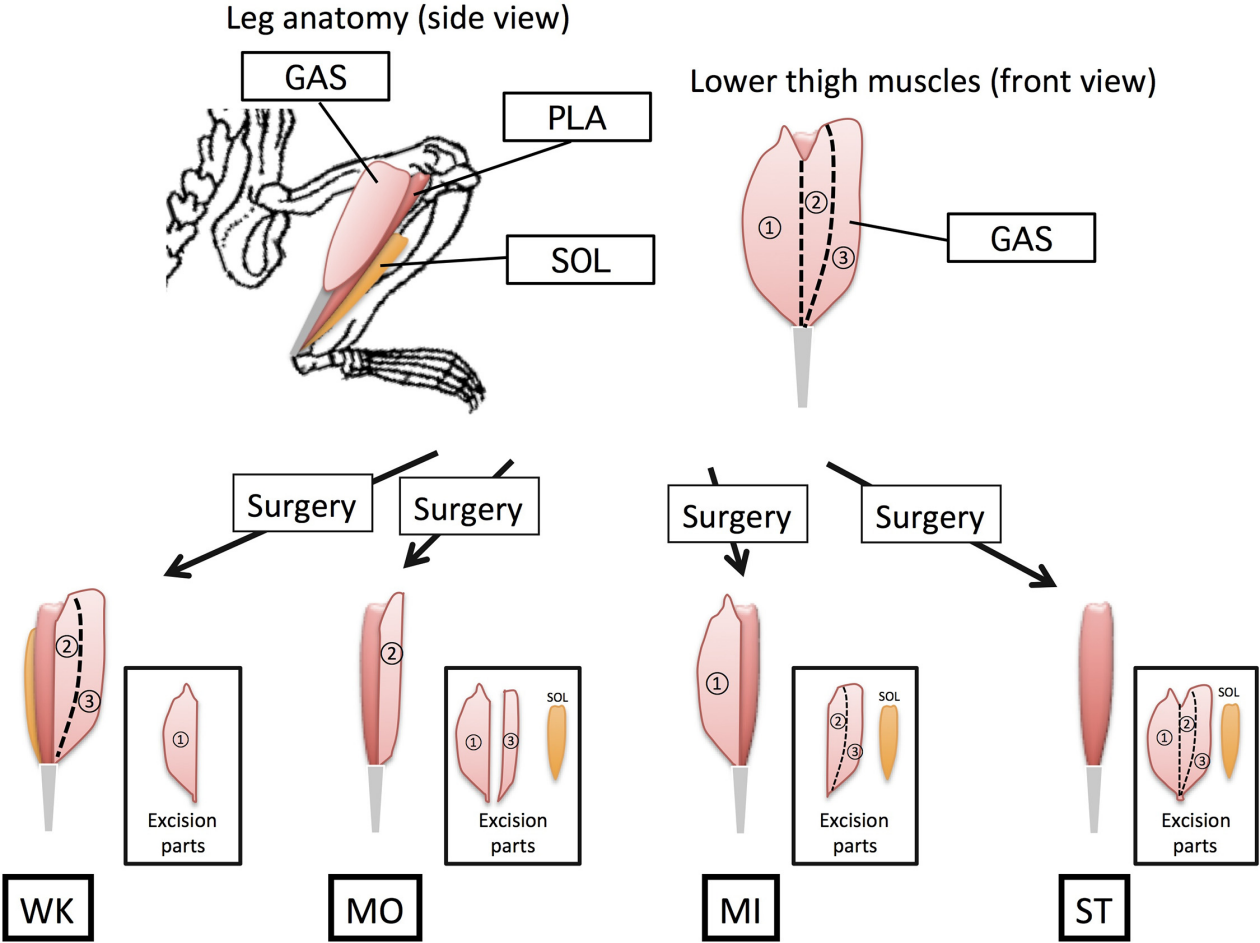
Partial-ablation surgery (schematic).

In all groups, rats were subjected to unilateral removal of the synergist muscles of the plantaris muscle under anesthesia (sodium pentobarbital, 60 mg/kg body weight, i.p.). The side of surgery was determined randomly, and the contralateral side was kept intact as the internal control (overloaded side (OL); Non-overloaded side (NL)). To control the magnitude of overload to the plantaris muscle, we altered the extent of surgical ablation of synergist muscle (Fig 1).

The ST group was subjected to ablation surgery, in which two major synergist muscles of the plantaris muscle, gastrocnemius (GAS) and soleus (SOL), were excised. In addition to this procedure, weaker overload was imposed on the plantaris muscle. In the WK group, the GAS muscle was cut longitudinally and its medial part removed; in the MO group, the medial and half of the lateral part of GAS and SOL muscles were removed; and in the MI group, the lateral part of GAS and SOL muscles were removed. During partial-ablation surgery, we ensured that the tendons and motor neurons of the GAS muscle remained intact to maintain the contractile function of the remaining part of the GAS muscle. In a preliminary experiment, the remaining parts of the GAS muscle were shown to be intact without any sign of atrophy, suggesting that they had normal function. After surgery, the incision was closed with a 4–0 nylon suture. Rats were returned to their cages and kept sedentary until sacrifice. To observe early responses of the plantaris muscle to overloading, rats were sacrificed 5 days after surgery (described as “5 days OL”; N = 8 in each group). Morphologic changes of muscle were investigated for rats sacrificed 14 days after surgery (“14 days OL”; N = 8 in each group).

### Muscle Sampling

Five days or 14 days after surgery, rats were sacrificed by an overdose of sodium pentobarbital (120 mg/kg body weight, i.p.). Samples of the plantaris muscle were taken immediately. Each muscle was weighed and cut transversely at its middle position. The distal half was soaked into optimal cutting temperature compound (Sakura Finetek, Tokyo, Japan), and then frozen quickly in isopentane chilled with liquid nitrogen. Samples were stored at –80°C until morphologic analyses. The proximal half was frozen quickly, powdered with liquid nitrogen, and stored at –80°C until biochemical analyses. Since the plantaris muscle weight is affected by the body weight[21], it was expressed relative to body weight (muscle weight / body weight).

### Cross-Sectional Analysis

Cross-sections (thickness, 10 μm) were cut from frozen samples of 14 days OL plantaris muscle and stained with hematoxylin & eosin (Sakura Finetek) according to standard procedures. Stained cross-sections were observed under a bright field microscope (Eclipse TE300; Nikon, Tokyo, Japan) and captured with a digital camera (DS-5M; Nikon, Tokyo, Japan). From photomicrographs of each muscle, cross-sectional area (CSA) of ≥1000 fibers was measured using ImageJ v. 1.46r (National Institutes of Health, Bethesda, MD, USA). The plantaris muscle has slow fibers in its deep region and fast fibers with different fiber size in its middle to surface region [22]. To avoid such a region-dependent bias, the measurement of CSA were made evenly for fibers in all captured images. As with muscle weight, fiber CSA is thought to be affected by body weight, so that it was expressed relative to body weight, too (CSA/body weight).

### Quantification of Ribosomal RNA (rRNA)

For rRNA quantification, total RNA was extracted from powdered samples of 5days OL using an RNA extraction kit (ISOGEN; Nippon Gene, Tokyo, Japan). Obtained RNA pellets were eluted with nuclease-free TE buffer (10 mM Tris-HCl, 1 mM ethylenediamine tetra-acetic acid (EDTA), pH 8.0). RNA concentration was measured with a spectrometer (Smart Spec; Bio-Rad, Hercules, CA, USA) at 260 nm and total RNA content in 1 mg of muscle determined. Total RNA solution equivalent to 125 μg of muscle tissue was electrophoresed on 1% agarose gel, stained with fluorescent dye (GRR-1000GR Red; Bio-Craft, Tokyo, Japan) and viewed under ultraviolet light. Using captured electrophoresis images, densitometric measurements were made for 18S rRNA and 28S rRNA using ImageJ. For direct comparison, samples from the eight experimental conditions (four groups × both legs) were run on the same gel.

### Western Blotting

Western blotting was undertaken according to the method described by Ogasawara et al. [23] with slight modifications. Powdered samples of 5 days OL were weighed and homogenized using shaking machine (Beads Crusher μT-12; Taitec, Koshigaya, Japan) with zirconia beads (3 mm × 3 pieces) in ice-cold homogenization buffer at 3,200 rpm for 1 min. The homogenization buffer contained 100 mM Tris-HCl (pH 7.8), 1% NP40, 0.1% sodium dodecyl sulfate (SDS), 0.1% sodium deoxycholate, 1 mM EDTA, 150 mM NaCl, protease inhibitor cocktail (Complete Mini; Roche, Basel, Switzerland), and phosphatase inhibitor cocktail (PhosSTOP; Roche). Homogenates were centrifuged at 15,000 × *g* for 15 min at 4°C and supernatants collected. Protein concentrations of supernatants were determined using a protein quantification kit (Protein Assay Rapid Kit; Wako Pure Chemical Industries, Osaka, Japan). Samples were mixed with ×3 sample buffer (1.0% *v/v* 2-mercaptoethanol, 4.0% *w/v* SDS, 0.16 M Tris-HCl (pH 6.8), 43% *v/v* glycerol, and 0.2% bromophenol blue) and heated at 95°C for 5 min. Samples with 50 μg of total protein were electrophoresed using polyacrylamide gel (gradient, 4%–20%), and transferred to polyvinylidene difluoride (PVDF) membranes. After transfer, membranes were washed with Tris-buffered saline containing 0.1% Tween-20 (TBST) and blocked with 5% skimmed milk in TBST for 1 h at room temperature. Membranes were then washed and incubated overnight with primary antibodies at 4°C. Primary antibodies (Cell Signaling Technology, Danvers, MA, USA) used were: phospho-p70S6 kinase (Thr389), total p70S6 kinase, and total ribosomal protein S6 (rpS6). After reactions with primary antibodies, membranes were washed and incubated with horseradish peroxidase-conjugated anti-rabbit antibodies for 1 h at room temperature. After washing, chemiluminescent reagents (Luminata Forte; Millipore, Bedford, MA, USA) were used to facilitate detection of protein bands. Images were scanned using a chemiluminescence detector (GeneGnome; Syngene, Cambridge, UK). After measurements, membranes were stripped using stripping buffer (Restore™ Plus; Pierce, Rockford, IL, USA) and stained with Coomassie Brilliant Blue (CBB) to verify equal loading in all lanes [24]. Using captured images, band intensities were measured using ImageJ. For direct comparison, samples from the eight experimental conditions were run on the same gel.

### Statistical Analyses

Data are the mean ± SEM. Results of body weights were analyzed by using two-way analysis of variance (ANOVA; Pre or Post- experiment × groups) with repeated measures on time. Following significant main or interaction effects, Sidak post hoc tests were used for pairwise comparison between time points and individual groups. For other variables, paired t-tests were used to comparing between NL and OL in each group. Differences between four groups’ NL legs or OL legs were determined by one-way analysis of variance (ANOVA) with Tukey’s post hoc testing. Pearson’s r product moment correlation coefficient was used to explore the relationship between different variables. *P* < 0.05 was considered significant.

## Results

### Changes in Body Weight

Table 1 displays changes of body weight pre and post experiment. There was not significant interaction between time and groups in both 5-day OL and 14-day OL (*P* = 0.29, 0.23, respectively). Body weight did not change significantly in any group after 5-day overloading (*P* = 0.14), but increased by ~12% after 14-day overloading in each group (*P* < 0.0001). After 14-day overloading, percentage changes in body weight were not significantly different between groups (*P* = 0.098).

**Table 1:** Body weight of 5 and 14day OL rats.

Group	*n*	Body weight, g		
		Pre-experiment	Post-experiment	% increase
		*5day*		
WK	8	355.4 sesei	361.5 sesei	1.76 sesei
MO	8	351.0 sesei	353.4 sesei	0.69 sesei
MI	8	386.5 sesei	385.1 sesei	-0.34 seseim
ST	8	355.9 sesei	357.0 sesei	0.37 sesei
		*14day*		
WK	8	332.4 sesei	375.4 sesei	12.86 seseim
MO	8	352.1 sesei	384.5 sesei	9.25 sesei
MI	8	327.3 sesei	366.6 sesei	12.02 seseim
ST	8	334.1 sesei	371.3 sesei	11.03 seseim

Values are means ± SEM; *n*, number of rats.

### Changes in Muscle Weight

**Fig 2:**
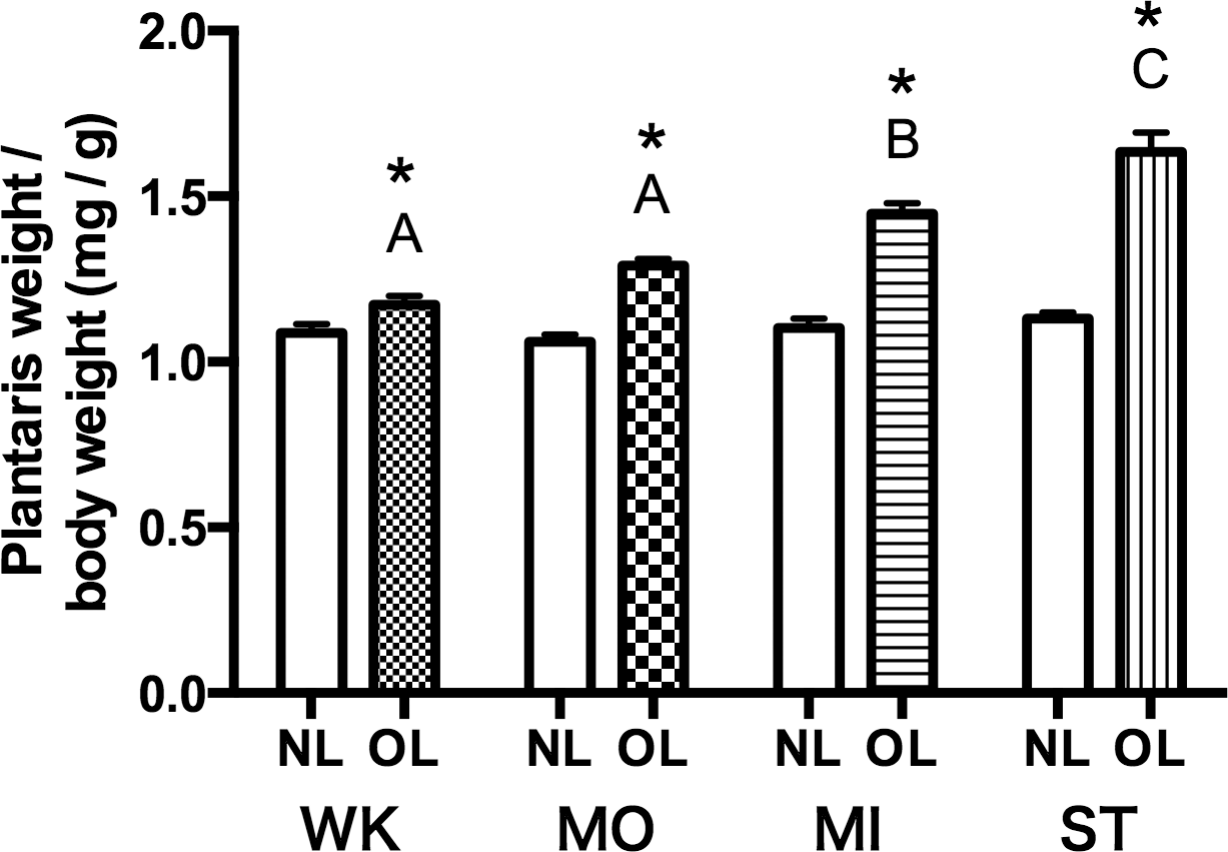
Plantaris muscle weight/body weight measured 14 days after surgery. Values are the mean ± SEM (N = 8). *Significantly different from NL in the same group (*P*<0.05). Different alphabetical letters denote significant differences between OL groups (*P* < 0.05).

Relative weight of the plantaris muscle (muscle weight/body weight) in 14 days OL rats is shown in Fig 2. In all groups, the relative weight of the plantaris muscle in OL legs was increased significantly compared with NL legs (increases of: 7.9 ± 2.4% in WK; 18.7 ± 2.0% in MO; 33.1 ± 3.0% in MO; 50.4 ± 5.3% in ST (*P* = 0.0004, *P* = 0.0003, *P* < 0.0001, *P* <0.0001, respectively)). Comparing between each group’s OL legs, there were significant difference between every groups (P < 0.05), excepting WK_OL vs. MO_OL (*P* = 0.14). These results suggest that surgical treatment caused graded increases in muscle weight. Relative weight of the plantaris muscle in NL legs between groups did not show significant differences (*P* = 0.22), suggesting that surgical treatment did not affect muscles in NL legs.

### Histological Changes

**Fig 3:**
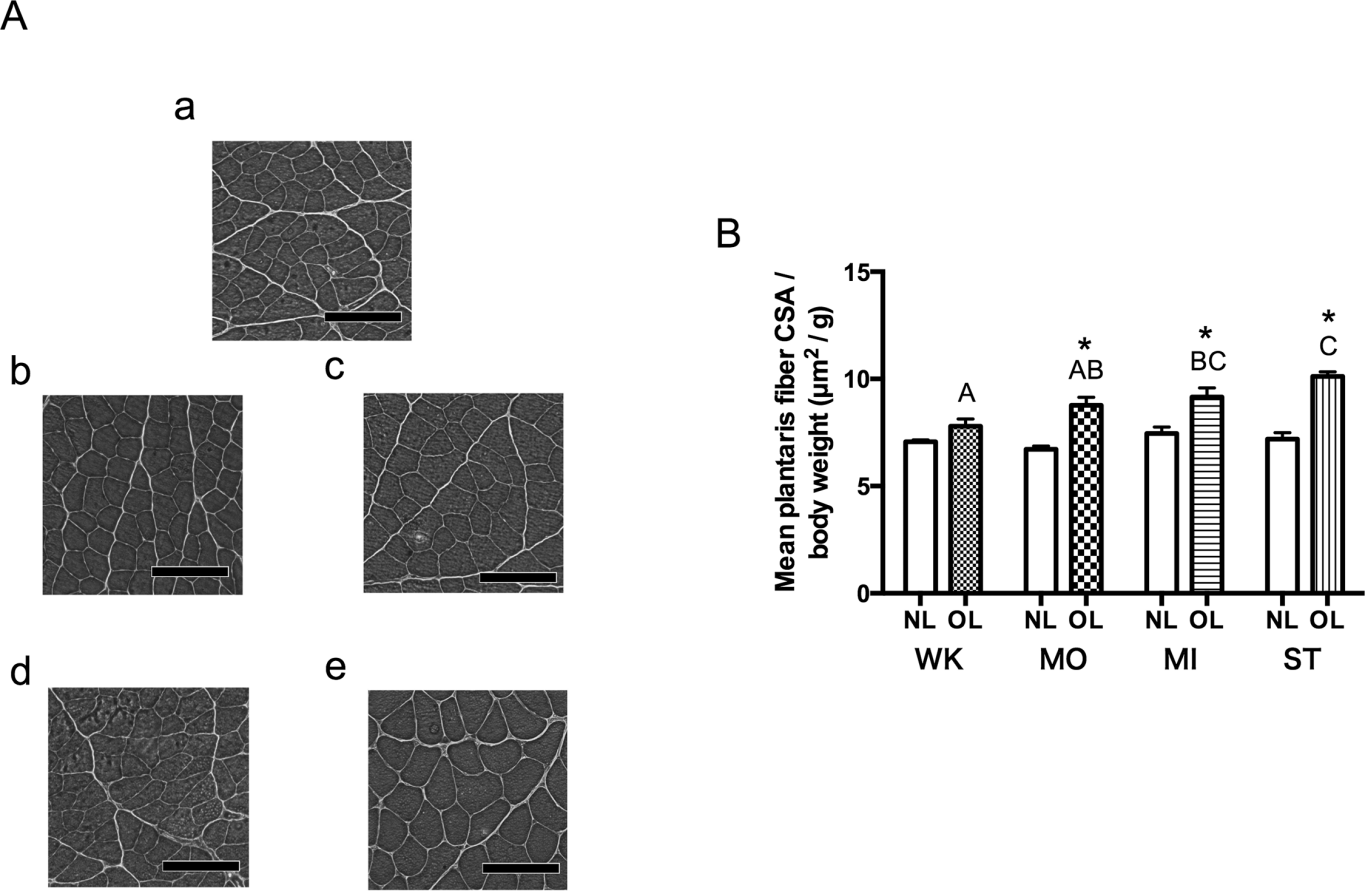
Morphologic analyses of the plantaris muscle obtained 14 days after surgery. A, Cross-sections of the plantaris muscle stained with hematoxylin & eosin. a, NL; b, WK_OL; c, MO_OL; d, MI_OL; e, ST_OL. Bar = 100 μm. B: Mean muscle fiber CSA/body weight. Values are the mean ± SEM (N = 8; measurements were made for ≥1000 fibers in each muscle). *Significantly different from NL in the same group (*P* < 0.05). Different alphabetical letters denote significant differences between OL groups (e.g., AB is different from C, but not different from A and B (*P* < 0.05)).

Typical photomicrographs of cross-sections of the plantaris muscle are shown in Fig 3A. In all four groups, muscle fibers exhibited hypertrophy without any sign of abnormality (e.g., edema, necrosis, apoptosis) (Fig 3A-b–e).

Absolute value of fiber CSA is shown in Table 2, and fiber CSA of fibers expressed relative to body weight is shown in Fig 3B. MO_OL, MI_OL, and ST_OL showed significant increases in relative fiber CSA compared with NL legs (increases of 29.0 ± 5.5% in MO_OL, 23.9 ± 3.9% in MI_OL, and 28.9 ± 2.2% in ST_OL compared with contralateral NL (*P* = 0.0011, *P* = 0.0005, *P* = 0.0001, respectively)), whereas in WK_OL, the change was not significant but tended to larger than WK_NL (*P* = 0.07). Comparing between each group’s OL legs, magnitude of the change in relative fiber CSA was significantly different between WK_OL *vs*. MI_OL (*P* = 0.045), WK_OL *vs*. ST_OL (*P* = 0.0003), and MO_OL *vs*. ST_OL (*P* = 0.049). Relative fiber CSA in NL legs between groups did not show significant differences (*P* = 0.18).

**Table 2:** Absolute value of fiber CSA (14day OL rats).

Group	*n*	NL	OL
WK	8	2651.8. Abs	2926.8. Abso
MO	8	2578.8. Abs	3366.8. Abso
MI	8	2724.7. Abs	3346.6. Abso
ST	8	2665.2. Ab.0	3759.8. Ab.0

Values are means ± SEM; *n*, number of rats.

### Ribosome Biogenesis

**Fig 4:**
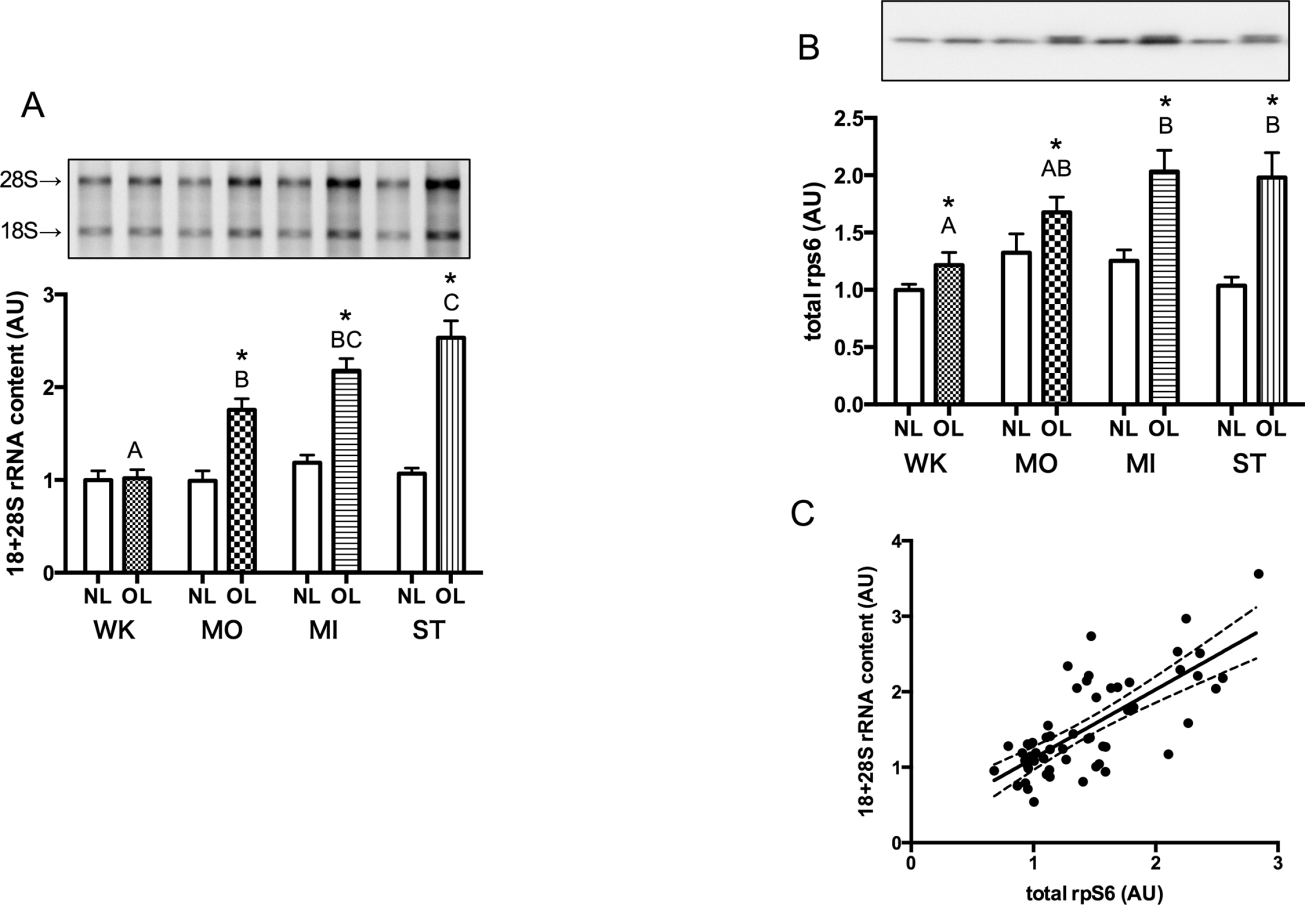
Content of 18+28S rRNA (A) and total rpS6 protein (B) in the plantaris muscle measured 5 days after surgery. C, Correlation between rRNA content and rpS6 content. Solid line represents least-squares linear regression (y = 0.9114x + 0.2064, *r* = 0.73, *P* < 0.0001), and broken lines denote its limits at 95% confidence intervals. Symbols and alphabetical letters are similar to those described in Figs 2 and 3.

The 18+28S rRNA content in a 1-mg sample of plantaris muscle, expressed relative to that in WK_NL, is shown in Fig 4A. In MO_OL, MI_OL, and ST_OL, it showed graded increases of 75.6 ± 12.1%, 117.9 ± 12.9%, and 153.5 ± 18.2%, respectively. Significant differences were seen in WK_OL vs. MO_OL, WK_OL vs. MI_OL, WK_OL vs. ST_OL, and MO_OL vs. ST_OL (*P* = 0.0032, *P* < 0.0001, *P* < 0.0001, *P* = 0.0018, respectively). These results suggested that rRNA content per muscle volume increased gradually with the increasing mechanical loading.

Total rpS6 content per unit weight of plantaris muscle is shown in Fig 4B. In all groups, total rps6 in OL legs was increased significantly compared with each NL legs (WK; *P* = 0.043, MO; *P* = 0.029, MI; *P* = 0.0004, ST; *P* = 0.0014, respectively). When the increase in 18+28S rRNA was plotted against the increase in rpS6, a strong correlation was found (*r* = 0.73, *P* < 0.0001, Fig 4C). The slope of least-squares regression was 0.911. Concomitant increases in rRNA and ribosomal protein suggested that ribosomal biogenesis is strongly stimulated by mechanical overloading of muscle.

### Phosphorylation of p70S6K

**Fig 5:**
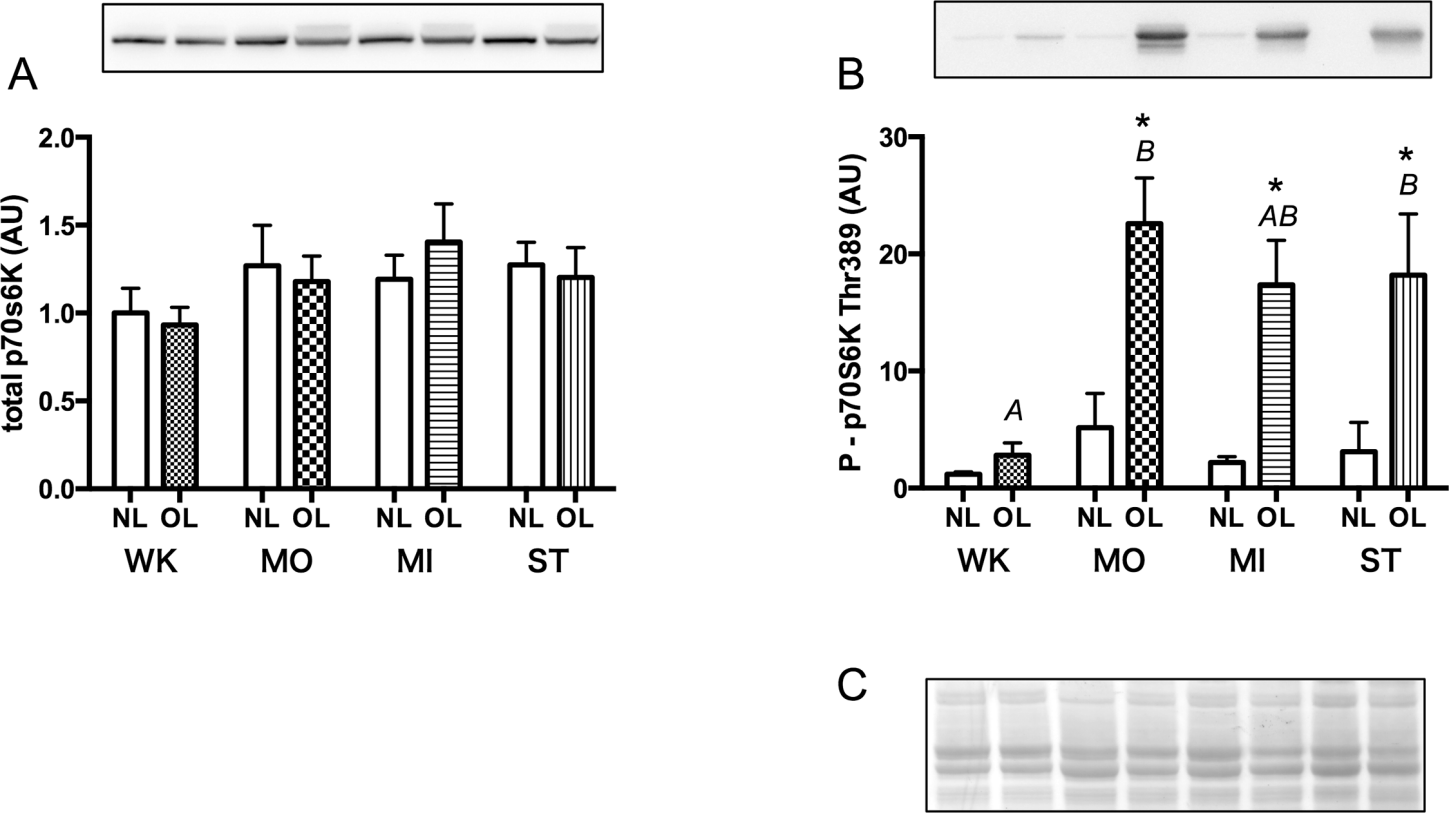
Content of total p70S6K (A) and its phosphorylated form (B) in the plantaris muscle measured 5 days after surgery. C, Representative CBB staining pattern of membranes: an almost identical amount of protein was loaded on electrophoretic lanes. Symbols and alphabetical letters are similar to those described in Figs 2 and 3.

Changes in expression of p70S6K and its phosphorylated form in the plantaris muscle are shown in Fig 5. CBB staining of PVDF membranes showed that an equal amount of total protein was loaded onto each electrophoresis lane (Fig 5C). Total content of p70S6K protein showed no significant differences between NL legs vs. OL legs in each group (WK; *P* = 0.18, MO; *P* = 0.51, MI; *P* = 0.25, ST; *P* = 0.24), or between groups in each leg (NL; *P* = 0.62, OL; *P* = 0.28) (Fig 5A).

However, p70s6K phosphorylation at Thr 389 in the muscles of OL legs showed a marked increase in the moderate loading condition (MO), but thereafter, no significant differences between MO, MI and ST groups were observed (MO_OL vs. MI_OL; *P* = 0.77, MO_OL vs. ST_OL; *P* = 0.85, MI_OL vs. ST_OL; *P* = 0.99) (Fig 5B). These results suggested that p70S6K phosphorylation readily reached a maximum and was kept almost constant even though the muscle size increased further with increasing mechanical loading.

### Relations between Muscle Weight and Contents of rRNA and Phosphorylated p70S6K

**Fig 6:**
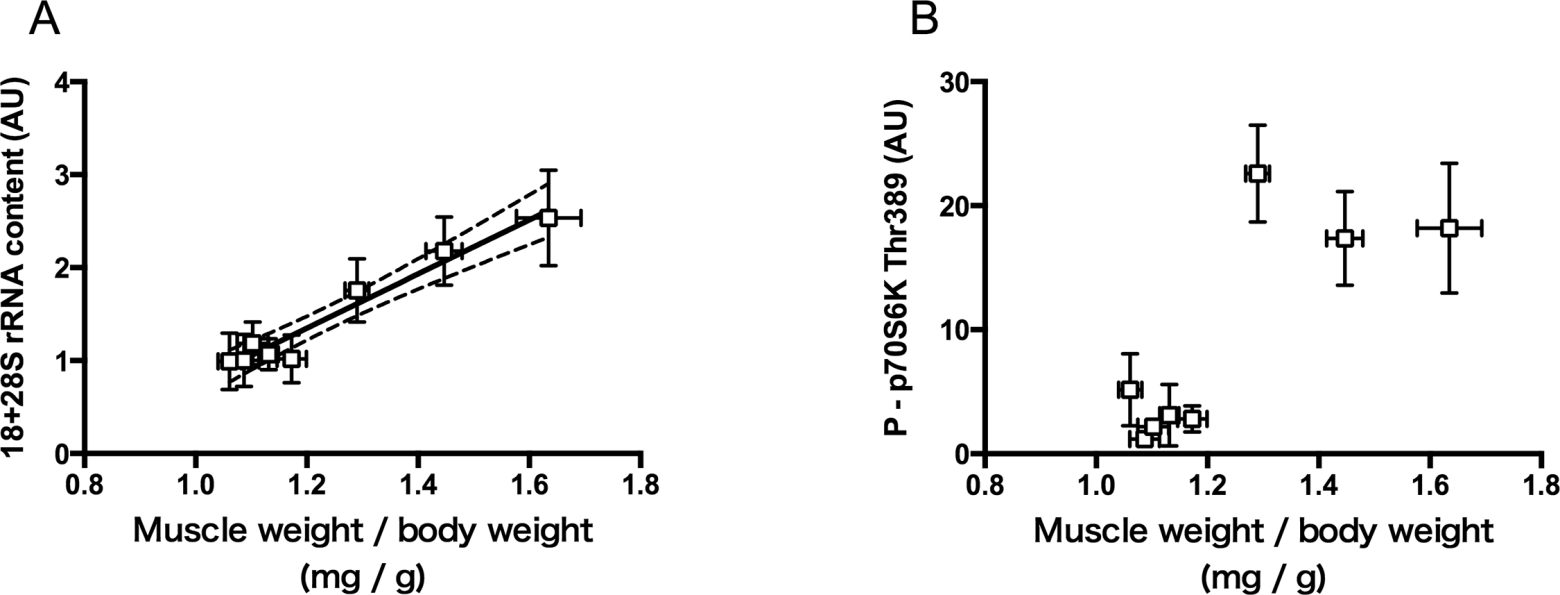
Relations between relative weight of the plantaris muscle and contents of 18+28S rRNA (A) and phosphorylated p70S6K (B). Relative muscle weight was measured 14 days after surgery, whereas levels of rRNA and phosphorylated p70S6K were measured 5 days after surgery (see Figs 4A and 5B). rRNA content is expressed relative to that in WK_NL. Mean ± SEM values are plotted. In A, the solid line represents least-squares linear regression (y = 3.171x − 215.3, *r* = 0.98, *P* < 0.0001), and broken lines denote its limits at 95% confidence intervals.

To obtain an insight into the significance of the early increases of rRNA and phosphorylated p70S6K in determining the magnitude of hypertrophy, contents of 18+28S rRNA and phosphorylated p70S6K measured at 5d after the surgery were plotted against the magnitude of hypertrophy measured at 14d after the surgery (Fig 6A and 6B). A strong linear relationship was found between relative muscle weight and 18+28S rRNA content (*r* = 0.98, *P* < 0.0001, Fig 6A).

On the other hand, the content of phosphorylated p70S6K showed a marked increase with relative muscle weight by up to ~20%, thereafter appeared to be almost constant despite of further increase in muscle weight (Fig 6B).

## Discussion

### Muscle Hypertrophy Caused by Synergist Ablation

Few studies have reported the relationship between the amount of mechanical stress and the magnitude of hypertrophy in overloading-induced muscle hypertrophy [17,25]. One of the major reasons for this is the difficulty in controlling the strength of mechanical overloading of muscle in animal models. Thus, we tried to develop a novel method of synergist ablation in which the amount of overloading of a given muscle can be controlled by partial removal of its synergist muscles (Fig 1).

The partial-ablation method described here caused plantaris muscle hypertrophy of varying magnitudes: 8%, 22%, 34% and 45%. Muscle fibers showed similar, graded increases in CSA. These results suggest that the strength of mechanical loading is one of the primary factors that determines the magnitude of muscle hypertrophy during a given time period. However, factors other than mechanical stress are also to be considered. For example, removal of the larger part of synergist muscles may give rise to the stronger stress in the nervous, endocrine and immune systems. In particular, increased inflammatory responses caused by synergist ablation surgery may produce a large amount of cytokines that may stimulate regeneration and /or growth of the subjected muscle.

### Ribosome Biogenesis

Previous studies have shown that ribosome biogenesis were activated in mouse muscles 7 days after synergist ablation [11] or serum-activated myotubes [26]. However, a quantitative relationship between ribosome biogenesis and the magnitude of muscle hypertrophy has not been reported.

We showed that expression of 18+28S rRNA, an indicator of ribosome content in the cell, increased significantly after synergist ablation, as has been shown in previous studies[11,14,27–30]. In addition, we found that rRNA content measured 5 days after surgery increased with the increase in relative muscle weight measured 14 days after surgery (Fig 4A). When rRNA content was plotted against the relative increase in muscle weight, a strong correlation was found (*r* = 0.98, *P* < 0.0001; Fig 6A), suggesting that activation of ribosome biogenesis at an early phase of overloading has an important role in increasing the translational capacity for subsequent muscle hypertrophy.

However, an appropriate amount of ribosomal protein commensurate with the increment in rRNA expression must be synthesized to assemble mature ribosomes. Chaillou et al. showed an increase in rpS6 (a component of the 40S ribosomal subunit) after synergist ablation [30]. We also showed that rpS6 content increased with an increase in muscle weight. In addition, a strong correlation was found between rRNA content and rpS6 content (r = 0.73, Fig 4C). A similar correlation between rRNA and expression of ribosomal protein has been reported for liver regeneration after hepatic resection, and is regarded as a reliable measure of ribosome biogenesis [31,32].

At present, it remains unclear whether ribosome biogenesis is necessary for attaining a large magnitude of muscle hypertrophy. Further studies on the relationship between ribosome biogenesis and protein synthesis, and on the effects of specific inhibitors of ribosome biogenesis are needed.

### Phosphorylation of p70s6K

Weigl demonstrated that p70s6K (a downstream protein in the mTOR signaling cascade) activates protein translation when phosphorylated at its Thr 389 residue at an early phase of mechanical overloading [33]. Some positive correlations have been reported between p70S6K phosphorylation and the magnitude of overloading-induced muscle hypertrophy, even though the range of hypertrophy was limited [17,34]. Therefore, the degree of p70s6K phosphorylation at an early phase of mechanical overloading may be a determinant of the magnitude of muscle hypertrophy [35]. However, more recent clinical studies of resistance exercise have shown weak or no correlation between p70S6K phosphorylation and the magnitude of hypertrophy [18–20].

The present study showed that a wide range of muscle hypertrophy (approximately 10%–50%) could be attained by controlling the degree of synergist ablation. A marked increase in p70S6K phosphorylation after moderate overloading was observed, a finding that was in accordance with other studies [11,27,28,30]. However, no further increase in p70S6K phosphorylation was observed with further increase in overload (Fig 5B). When phosphorylation level was plotted against the magnitude of muscle hypertrophy, it was found to reach a maximal phosphorylation level at ~20% hypertrophy, which remained almost constant thereafter (Fig 6B).

The results of the present study suggest that p70S6K phosphorylation can regulate only a small rate of muscular growth, and that an increase in translational capacity (i.e., ribosome biogenesis) is required to gain a larger magnitude of hypertrophy. However, sustained overloading of muscle by synergist ablation may cause a much stronger stimulus than in physiological interventions such as electric stimulation [17] and resistance exercise [34]. Therefore, the exact role of ribosome biogenesis in muscular adaptation to resistance training at a much prolonged period of time is not clear. Further studies with an animal model of exercise [23,36] must be conducted for ribosome biogenesis and activation of the mTOR signaling cascade.

## Conclusion

In conclusion, the present study with a modified synergist ablation method showed that increase in ribosome biogenesis at an early phase of compensatory muscle hypertrophy was strongly correlated with the magnitude of hypertrophy, suggesting that it plays an important role in increasing the translational capacity for sustained protein synthesis. Further studies are required to clarify the role played by ribosome biogenesis in exercise-induced muscle hypertrophy.

## Supporting Information

S1 Dataset The dataset of sample groups used in the analysis. (XLSX)
